# The protective effect of various forms of *Nigella sativa* against hepatorenal dysfunction: underlying mechanisms comprise antioxidation, anti- inflammation, and anti-apoptosis

**DOI:** 10.3389/fnut.2025.1553215

**Published:** 2025-05-13

**Authors:** Reham M. Algheshairy, Hend F. Alharbi, Mona S. Almujaydil, Raghad M. Alhomaid, Hoda A. Ali

**Affiliations:** ^1^Department of Food Science and Human Nutrition, College of Agriculture and Food, Qassim University, Buraydah, Saudi Arabia; ^2^Department of Nutrition and Clinical Nutrition, College of Veterinary Medicine, Cairo University, Cairo, Egypt

**Keywords:** antioxidant, apoptosis, cytokine, cytokeratin-18, hepatorenal, *Nigella sativa* oil, *Nigella sativa* seed

## Abstract

**Introduction:**

The liver and kidney are vital organs that are interconnected, dealing with detoxifying and excreting xenobiotics. They are constantly exposed to oxidative stress, which can cause hepatorenal dysfunction. This study compares two forms of *Nigella sativa* (NS), NS oil (NSO), and NS seeds (NSS), for the first time, in their ability to mitigate hepatorenal injury induced by azathioprine (AZA), exploring potential underlying mechanisms.

**Methods:**

Group (1): negative control; Group (2): positive control received 15 mg/kg AZA orally. Groups (3, 4, and 5) received 100 mg/kg silymarin (standard reference), 500 mg/kg NSO, and 250 mg/kg NSS, respectively, and were subjected to the same dose of AZA. A one-way analysis of variance was conducted, followed by Mann-Whitney *post-hoc* analysis.

**Results:**

Administration of AZA induced hepatorenal dysfunction, evidenced by dyslipidemia, elevations in serum liver enzymes, creatinine, urea, pro-inflammatory cytokines, and cytokeratin-18. Antioxidant enzymes in liver and kidney tissues were reduced, with an elevation in caspase-3 and caspase-9. Both forms of NS significantly balanced serum pro- inflammatory cytokines (14.33 ± 2.33, 15.15 ± 1.64 vs. 24.87 ± 1.87) pg/ml, interleukin-4 (16.72 ± 1.14, 15.95 ± 1.03 vs. 10.64 ± 1.04) pg/ml, and interleukin-10 (19.89 ± 0.69, 18.38 ± 0.38 vs. 15.52 ± 1.02) pg/ml, and downregulated cytokeratin-18 (210.43 ± 21.56, 195.86 ± 19.42 vs. 296.54 ± 13.94) pg/ml for NSO and NSS vs. the positive group, respectively. NSS enhanced liver antioxidant activity (*P* < 0.05), normalized liver enzymes (*P* < 0.05, *P* < 0.01) for alanine aminotransferase and aspartate aminotransferase, respectively, and significantly lessened dyslipidemia (*P* < 0.05). Liver caspase-3 and caspase-9 improved significantly with NSS, while kidney caspase-3 and caspase-9 improved with NSO. NSO increased kidney glutathione peroxidase and catalase (*P* < 0.01) and corrected creatinine and urea (*P* < 0.05). Histopathological observations confirmed the present data.

**Discussion:**

Conclusively, NSO and NSS mitigated hepatorenal dysfunction responses to AZA through antioxidant, anti-inflammatory, and anti-apoptosis properties that underlie their protective performance. Interestingly, NSO surpassed NSS in restoring renal oxidative damage, while NSS provided better hepatic protection than NSO, suggesting NSO for patients with kidney dysfunction and NSS for those with liver problems.

## 1 Introduction

Recent modern lifestyles and environmental pollution have originated from huge industrialization. The toxicity of many toxic chemicals has become a significant public health hazard worldwide, inducing oxidative stress in multiple organs, particularly the liver and kidney (1). Various medications, such as chemotherapies, can inhibit purine metabolism, leading to DNA damage (2) and destroy biological proteins and lipid membranes, producing a variety of pathological alterations such as hepatorenal damage (3). In addition, toxic metals and chemicals from frequently used utensils stored in tissues may trigger oxidative stress and induce toxicity in various organs, such as hepatotoxicity and nephrotoxicity (4). Oxidative stress can damage most glomeruli and tubules in the kidney (5) and cause hepatic disease (6) due to lipid peroxidation, which may lead to liver cell necrosis.

The liver and kidney are essential organs that are interdependent, involved in body metabolism, detoxifying environmental pollutants and harmful chemicals, and excreting xenobiotics (7, 8). Yearly, almost 2 million cases of morbidity and mortality globally are attributed to liver disorders (9), while about 11.3 million incidences of renal dysfunction are estimated in the developing world (10). Finding effective treatments for these two efficient organs with the least side effects is important. Most therapeutic options for liver and kidney injuries are unreliable (11). A new medication that can restore and prevent liver or kidney damage safely and efficiently remains challenging (7). Therefore, it is critical to find effective naturally medicated plants for the prevention and treatment of liver and kidney liver diseases such as *Nigella sativa* (1, 12). Medicinal plants have been employed to prevent and treat liver diseases (13). Black seed *(Nigella sativa L.)* belongs to the *Ranunculaceae* family and typically grows in Mediterranean regions, the Middle East, Western Asia, and Eastern Europe (14, 15). Historically, it has been used as a traditional medicinal plant to remedy many illnesses, including headaches, cough, flatulence, uricosuric antispasmodic, and choleretic. *Nigella sativa* has widespread pharmacological and therapeutic effects, including anticancer, antioxidant, hepatoprotective (16), anti-inflammatory (17), and renal protective activities (18). The bioactive components of black seed have made it a focus for research and an antidote for hepatorenal dysfunction Nevertheless, the curative effects of Nigella sativa depend on its form. For instance, the seed and oil forms are used to treat disorders of the nervous, cardiovascular, respiratory, digestive, and immune systems (19), while Nigella sativa seeds are used as analgesics, immunomodulatory agents, anti-histaminic, and anti-leukotriene agent (20). Hepatotoxicity and renal toxicity tend to be alleviated by *Nigella sativa* seed extract (7, 21). *Nigella sativa* supplementation has been shown to reduce hepatorenal injury caused by Amikacin in rabbits (12). Numerous studies have demonstrated the preventive and antioxidant properties of NSO against drugs and chemical hazards from xenobiotics (22, 23). Supplementation with Nigella sativa seeds can reduce aluminum toxicity and normalize liver and kidney functions (5). Likewise, *Nigella sativa* seeds have protected the nephrotoxicity induced by several anti-inflammatory agents (24). Patients suffering from non-alcoholic fatty liver disease have benefited from treatment with *Nigella sativa* seeds (25). A dietary supplement containing *Nigella sativa* powder has shown antioxidant capacity and modulation of *Burkholderia cepacia* infection in Nile tilapia (26). Conversely, there was no significant effect on liver enzymes (AST and ALT) after treatment with *Nigella sativa* in rats (27).

Azathioprine is a chemotherapeutic, antimetabolic, and immunosuppressive agent that acts as an antagonist of purine metabolism (28), with considerable risks associated with organ toxicity, particularly to the liver and kidney (29). In contrast, silymarin is an extract from milk thistle seeds and is effective in improving hepatic (30) and renal disorders (31). The current study used azathioprine to induce hepatorenal damage, with silymarin serving as a standard reference for liver and kidney functions.

Although various forms of *Nigella sativa*: extract, oil, seed, and powder have been evaluated separately by previous researchers as antidotes for hepatorenal toxicity, no one *has* compared their potential protective effects on hepatorenal dysfunction. In addition, the unclear interpretation of the mitigation mechanism of *Nigella sativa* in hepatorenal injuries remains unexplained. These limitations prompted the current work to compare the most popular forms of *Nigella sativa*: *Nigella sativa* oil and *Nigella sativa* seed for the first time as a natural intervention to answer the prevalent question of which form offers more potential protection against hepatorenal complications. This study explores the underlying potential mechanisms that protect against hepatorenal complications, including antioxidative, anti-inflammatory, and anti-apoptotic properties.

## 2 Materials and methods

### 2.1 Ethical standard

Under approval number “23-24-23,” this study was permitted by the Committee of Health Research Ethics at Qassim University, Kingdom of Saudi Arabia, according to the International Animal Ethics Committee.

### 2.2 *Nigella sativa* forms (oil and seed)

*Nigella sativa* oil (NSO) was purchased from the International Zamzam Company for natural oils, herbs, and cosmetics, London, UK, under a license from the Ministry of Health in Cairo, Egypt. It was cold*-*pressed to ensure maximum quality, without any additives or preservatives. The raw organic *Nigella sativa* seed (NSS) was obtained from iHerb, Kevala, USA; the black seeds are products of Egypt and were packed in the USA.

### 2.3 Total phenolic and total flavonoid compounds of NSS, NSO

Spectrophotometer, UV/Vis, Jenway, England, was used under environmental conditions: temperature of 20°C and 37% rH humidity. Total phenols were determined colorimetrically using the Folin–Ciocalteu reagent. Utilizing the regression equation of the standard plot (y = 1,001.1x + 4.4832, r^2^ = 0.9993), the total phenolic content was expressed as mg gallic acid equivalent/kg sample (32). The aluminum chloride colorimetric technique was used to calculate the total flavonoid content (33). The following were added: 5.6 ml of distilled water, 0.2 ml of 10% aluminum chloride, 0.2 ml of 1M potassium acetate, and 3 ml of methanol with 1 ml of sample extract, which was allowed to stand for 30 min at room temperature. The absorbance was measured at 420 nm. Standard doses of rutin (1 mg/ml) were employed. The total flavonoid content was computed and reported as mg Rutin equivalent/100 g sample, utilizing the regression equation of the standard plot (y = 372.82x−4.2562, r^2^ = 0.996).

### 2.4 Antioxidant activities of NSS and NSO

An assay method by Brand-Williams et al. (34) and UV/Vis Spectrophotometer (*Jenway*, England) was employed to measure 2,2-diphenyl-1-picrylhydrazyl (DPPH) at 24°C and 32.3% rH. Methanol was used to prepare concentrations from 2/100 gm to 10/100 gm from a sample. DPPH radical (100 μL, 0.2 mM) and extract (100 μL) were dissolved in methanol. After stirring, the mixture was left in the dark for 15 min. The absorbance was then assessed at 517 nm in comparison to a blank. The test was conducted at 25°C with 38% rH. The percentage scavenging effect was computed using the formula [(Ao – A1)/Ao] × 100, where Ao is the absorbance without the sample and A1 is the absorbance with the sample.

### 2.5 High-performance liquid chromatography

The process described in Agilent Application Note Publication 5991-3801EN, 2014, was used. The column employed was a Kinetex^®^ 1.7 μm EVO C18 50 mm x 2.1 mm (Phenomenex, USA) with a quaternary pump equipped with the Agilent 1260 Infinity HPLC Series (Agilent, USA). The temperature was set at 30°C. A ternary linear elution gradient was utilized for separation, comprising (A) HPLC grade water with 0.1% *H3PO4* (v/v), (B) acetonitrile with 0.1% *H3PO4* (v/v), and (C) methanol, with a flow rate of 0.2 ml/min. A volume of 20 μl was injected. Detection was performed using a variable wavelength detector adjusted to 280 nm, at 20°C and 38% rH. Retention time and UV spectra were used to identify the phenolic compound peaks, which were then compared to standards (35).

### 2.6 Silymarin (*Silybum marianum*)

Silymarin standardized to 280 mg Silymarin Flavonoids—equivalent 80%—was obtained from Botanicals/Herb NOW FOODS (395 S. Glen Ellyn Rd., Bloomingdale, IL 60108, USA; nowfoods.com).

### 2.7 Hepatorenal damage induction

Azathioprine (AZA), under the commercial name Imuran, was selected for this investigation to induce hepatorenal damage. The AZA was obtained as a 50 mg tablet (EXCELLA GmbH & Co.; Aspen Pharma Trading Limited, Citywest, Dublin, Ireland KG, Germany). For 30 days, rats were administered 15 mg/kg body weight/day AZA per os in a saline solution to induce hepatorenal injury (36).

### 2.8 Animals

Healthy adult Wistar rats, around 7 weeks of age and weighing approximately 180 ± 11 gm, were purchased from the animal husbandry section of the laboratory center at King Saud University, Riyadh, Saudi Arabia. The animals were transferred to a suitable room for rearing at Qassim University, KSA, in the College of Agriculture and Veterinary Medicine's Department of Food Science and Human Nutrition. All the rats were housed in a wire battery, five rats per cage, for 1 week before the start of the experiment for acclimatization to the experimental room conditions. The rearing room was adjusted for optimal conditions: temperature (23°C ± 2), relative humidity (55% ± 5%), and a photoperiod (12 h light followed by 12 h dark cycle). A basal commercial diet and fresh water were offered to the animals *ad libitum* throughout the study. The commercial diet was supplied by the General Company of Feed Mills, which met the nutrient requirements based on the National Research Council's recommendations (37). The current experiment adhered to the rules and ethics for animal care recommended by the Deanship of Scientific Research at Qassim University.

### 2.9 Experimental design and protocol

Forty rats were grouped into five groups of eight animals each. Prior to the experiment, the animals in each group were weighed. The groups were given different treatments: the first (negative control) was administered saline solution through gavage. The second (positive control) was given the same as the first group for 14 days. Groups 3, 4, and 5 were offered 100 mg/kg body weight silymarin (38), 500 mg/kg body weight of NSO (39), and 250 mg/kg body weight NSS(40), respectively, for 42 days. On day 15, all groups except the first one were given 15 mg/kg body weight/day AZA (36) in a saline solution. Over 30 days, the rats in the treatment groups received both the supplements and AZA simultaneously. The doses of silymarin and NSO were administered daily by gavage, whereas NSS was crushed to a fine powder and mixed with the diet at a dose of 2.5 g/kg (calculated to correspond to 250 mg/kg body weight). The doses of AZA and the other supplements (silymarin, NSO, and NSS) were calculated based on the equivalent human dose consumption according to the previously proposed conversion equation (41). Assuming that the AZA treatment dose is 1–4 mg/kg for humans, the recommended dose of silymarin is 15–20 mg/kg, and a human takes one teaspoonful of NSO daily, along with 2–3 g/day of NSS. Fortunately, most of the calculated doses used in this study align with those suggested in many previous studies.

Rats were weighed weekly to calculate the precise amount of each supplement. At the end of the experiment, blood was extracted from the rats through ocular techniques, and serum was collected after centrifugation for 10 min at 2,000 x g. The serum was stored at −20°C in a deep freezer for biochemical analysis, including lipid profile, liver function tests, kidney function tests, inflammatory cytokines, and cytokeratin 18.

Three animals were randomly chosen from each group and anesthetized before being sacrificed. The liver and both kidneys were gently removed, rinsed in ice-cold 1.15% KCl, and placed on filter paper. The liver and left kidney were weighed. The liver was divided into two portions; one section of the liver and one kidney were used to determine antioxidant activities, lipid peroxidation, and apoptotic enzyme activities. The other kidney and the second section of the liver were used for histological observations.

### 2.10 Liver and kidney homogenization

One section of the liver and one kidney were individually prepared for homogenization according to the manufacturer's guidelines. Briefly, either the liver portion or the kidney tissue specimens were combined with PBS (pH 7.4) and centrifuged for 20 min at 1,200 g at 4°C to obtain the supernatant fluid (42). The Lowry method (43) was used to measure the protein concentration of the supernatant. The supernatants collected from the liver and kidney were kept separately for assays of antioxidant activities, lipid peroxidation, and apoptotic enzyme activities.

### 2.11 Relative liver and kidney weights

After being cleaned in a standard saline solution, the left kidney and liver were weighed individually on a sensitive balance to determine their absolute weights. The body weight (g) ratio was utilized to calculate each organ's relative weight. Relative organ weight = absolute weight of organ/body weight **×** 100 (44).

### 2.12 Lipid profile

Serum triglyceride, total cholesterol, and HDL levels were estimated using commercial colorimetric kits from Biodiagnostic, Diagnostic, and Research Reagents, Cairo, Egypt. Meanwhile, LDL was calculated using the formula: LDL = (Total Cholesterol) – (HDL) – (TGs/5) (45).

### 2.13 Liver and kidney function tests

Serum alanine aminotransferase enzyme (ALT), aspartate aminotransferase enzyme (AST), creatinine, urea, Na, and K were measured using the colorimetric method with commercial kits specifically designed for laboratory use from Biodiagnostic, Diagnostic, and Research Reagents, Cairo, Egypt.

### 2.14 Inflammatory cytokines

Using the ELISA (SUNLONGBIOTETECH, Co., Ltd., China), tumor necrosis factor α (TNF-α) was measured, catalog number (SL0722Ra). The sensitivity was 2.8 ng/mL, with intra-assay and inter-assay coefficients of variability (CVs) of <10.5% and 12.8%, respectively (SUNLONGBIOTETECH, Co., Ltd., China) catalog number (SL0409Ra) was applied to detect the anti-inflammatory cytokines, interleukin 4 (IL-4). The sensitivity was 0.1 pg/mL, the intra-assay and inter-assay CVs were <10.2% and 12.5%, respectively. ELISA (SUNLONGBIOTETECH, Co., Ltd., China) was implemented to detect the interleukin 10 (IL-10) catalog number (SL0415Ra). The intra-assay CV and inter-assay CV were (10.7% and 12.6%), respectively, and sensitivity was 0.5 pg/mL.

### 2.15 Cytokeratin 18

Using the ELISA (SUNLONGBIOTETECH, Co., Ltd., China) catalog number (SL1216Ra), cytokeratin 18 was detected. The intra-assay CV was <10.4%, whereas the inter-assay CV was 12.6%. The sensitivity was 12 pg/mL.

### 2.16 Antioxidant activities and lipid peroxidation of liver and kidney tissues

Kits made for laboratory purposes were purchased from Biodiagnostic, Diagnostic, and Research Reagents in Cairo, Egypt, to determine the antioxidant enzyme activity of glutathione peroxidase (GHPx) and superoxide dismutase (SOD) concentrations in both liver and kidney tissues (catalog numbers GP 25 24 and SD 25 21, respectively). A kit from Elabscience, USA (catalog number M-BC-K031-S), was used to assess catalase activity (CAT). Additionally, the kit from Biodiagnostic, Diagnostic, and Research Reagents in Cairo, Egypt (catalog number MD 25 29), was employed to determine lipid peroxidation (MDA).

### 2.17 Apoptotic enzyme activities of liver and kidney tissues

Caspase-3 and caspase-9, which are apoptotic enzymes, were selected for detection in this study. Using ELISA, caspase-3 activity was measured with a kit from SUNLONGBIOTETECH, Co., Ltd., China (catalog number (SL0152Ra). The sensitivity was 0.01 ng/mL, while the intra-assay and inter-assay CVs were <10.6% and 12.4%, respectively. The ELISA was used to determine the activity of caspase-9 (SUNLONGBIOTETECH, Co., Ltd., China) catalog number (SL0154Ra). The sensitivity was 0.025 ng/mL, while the intra-assay and inter-assay CVs were <10.8% and 12.7%, respectively.

### 2.18 Histopathological observation

The specimens of the liver and remaining kidney, previously preserved for histological examination, were fixed in 10% formal saline and processed using routine paraffin wax techniques for histopathological purposes. Hematoxylin and eosin (H and E) staining was employed for the tissues (46).

### 2.19 Statistical analysis

Means of the standard errors were used to represent the data values. For each measured parameter, a basic one-way analysis of variance (ANOVA) test was conducted. *Post hoc* analysis and the Mann–Whitney test were used to compare the negative and positive control groups. Furthermore, a significant difference was observed when comparing the positive group with the supplemented groups (NSO and NSS) and the silymarin standard drug group against the NSO and NSS groups, with *P* < 0.05 and *P* < 0.01, reflecting statistical significance.

## 3 Results

### 3.1 Total phenols, total flavonoids, and antioxidant activity of NSO and NSS

The findings illustrated in Table 1 reveal that both sources of NS (oil and seeds) possess considerable quantities of total phenols and total flavonoids. However, NSO contained more phenols and total flavonoids than NSS (170.80 ± 12.8, 591.00 ± 30.6 vs. 137.98 ± 11.7, 376.33 ± 28.5). The antioxidant activity of NSO and NSS, represented by DPPH at different percentages (0.10, 0.25, and 0.50), indicated that both NSO and NSS exhibit antioxidant properties. Nevertheless, NSO demonstrated greater antioxidant power than NSS (76.75 ± 10.4, 89.79 ± 9.5, 91.21 ± 11.6 vs. 31.13 ± 7.8, 61.17 ± 8.7, and 79.50 ± 8.3). A positive relationship was observed between the increase in concentration and the percentage of DPPH radical scavenging activity for both NSO and NSS.

**Table 1:** Total phenols, total flavonoids, and antioxidant activity of NSO and NSS.

***Nigella sativa* form**	**Total phenols (mg gallic acid equivalent/kg)**	**Total flavonoids (mg rutin equivalent/100g)**	**% DPPH radical-scavenging activity**		
			**0.10%**	**0.25%**	**0.50%**
NSO	170.80 ± 12.8	591.00 ± 30.6	76.75 ± 10.4	89.79 ± 9.5	91.21 ± 11.6
NSS	137.98 ± 11.7	376.33 ± 28.5	31.13 ± 7.8	61.17 ± 8.7	79.50 ± 8.3

Means ± standard error (SE). DPPH, 2,2-diphenyl-1-picrylhydrazyl expresses percentage inhibition of the DPPH radical-scavenging activity NSO, *Nigella sativa* oil; NSS, *Nigella sativa* seeds.

### 3.2 Bioactive phytochemical constituents of NSO and NSS detected by HPLC

**Figure 1:**
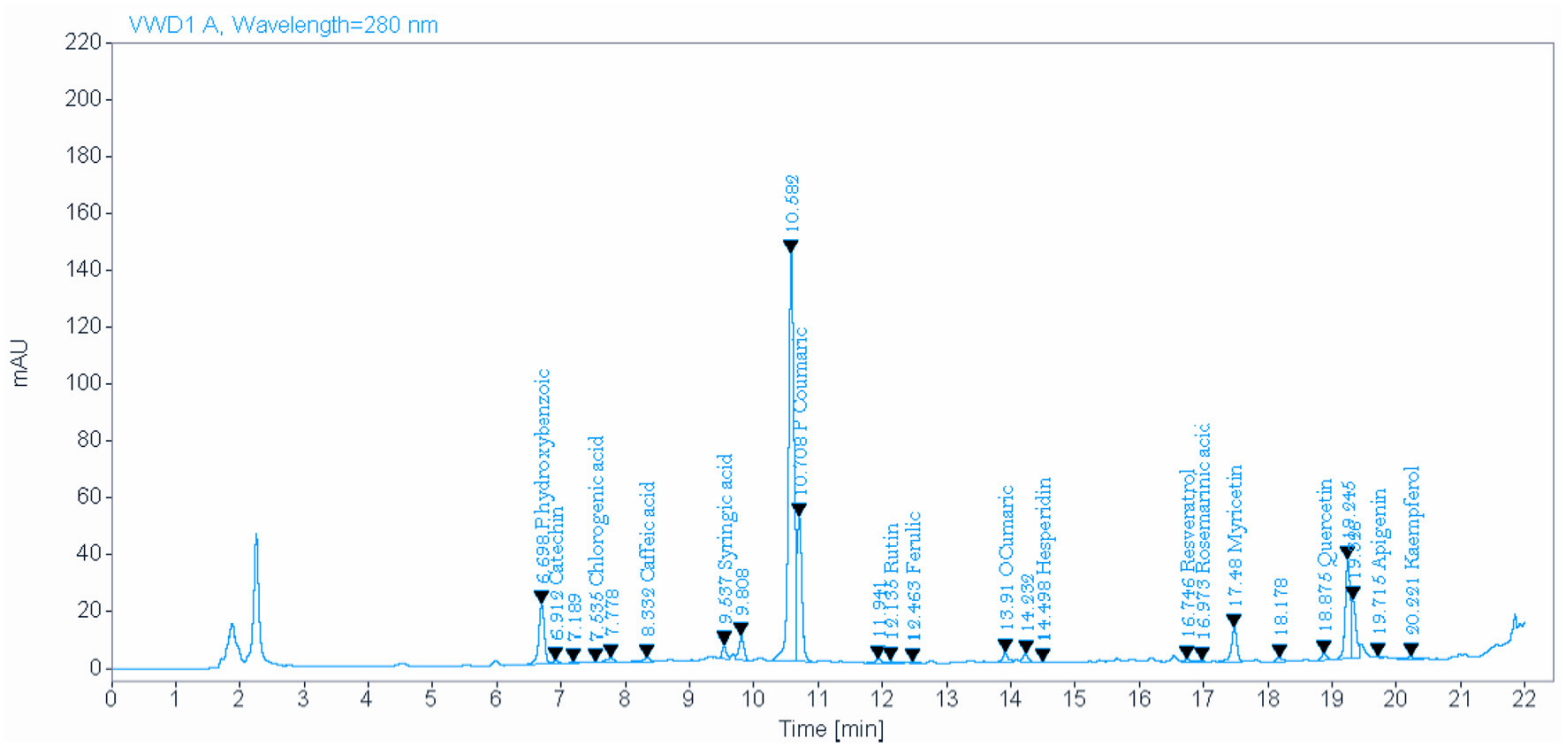
Identify and quantify some bioactive phytochemical constituents of *Nigella sativa* oil detected by HPLC.

**Figure 2:**
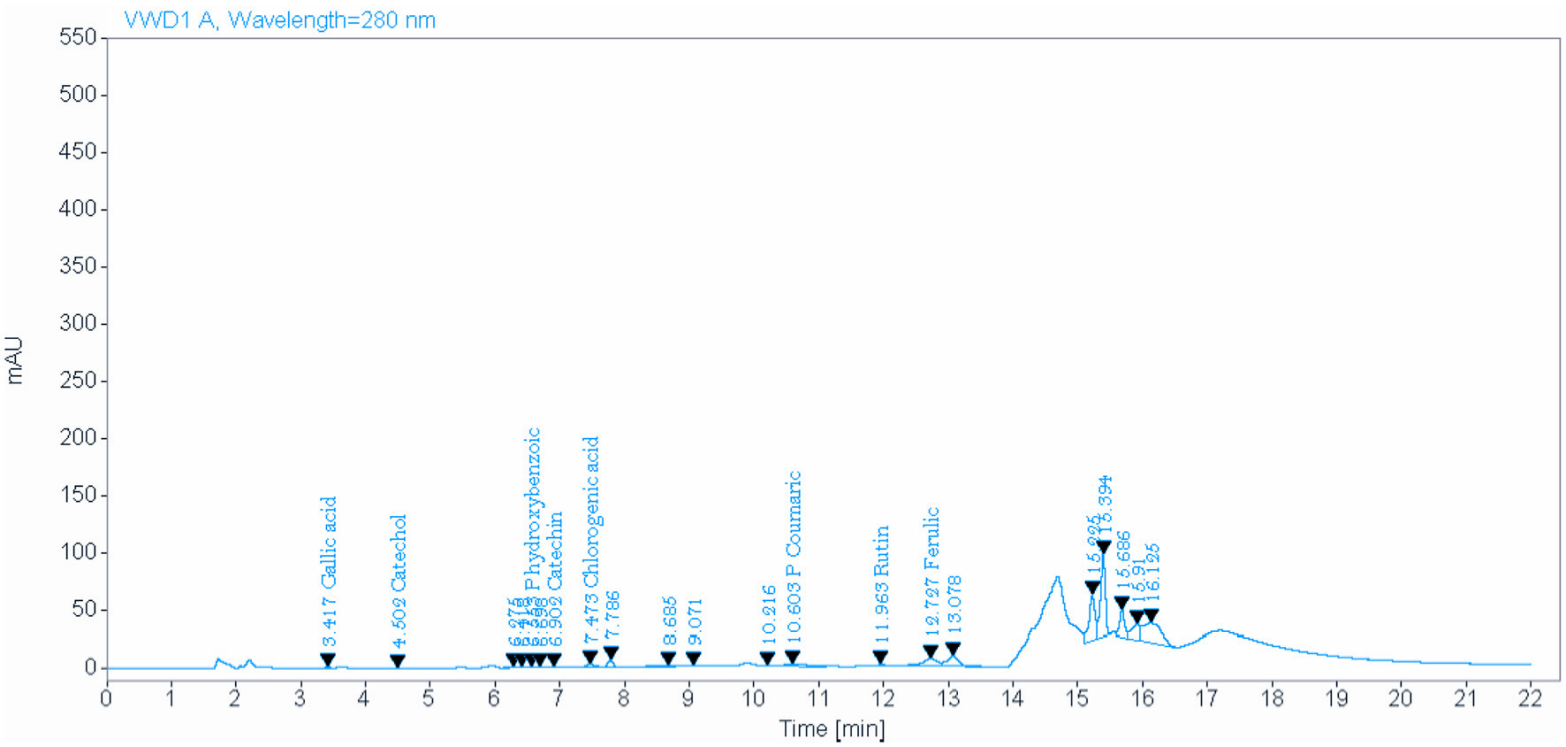
Identify and quantify some bioactive phytochemical constituents of *Nigella sativa* seed detected by HPLC.

The analysis of NSO using the HPLC method showed the presence of various bioactive phytochemical constituents. However, the highest amounts detected in NSO were 33.096, 18.420, and 15.858 mg/kg for P-hydroxybenzoic, P-coumaric, and quercetin, respectively (Figure 1, Table 2). Interestingly, the bioactive phytochemical components recorded in NSS were significantly different from those in NSO. The major content detected in NSS was ferulic acid, which constituted the highest amount at 41.987 mg/kg (Figure 2, Table 3).

**Table 2:** Identify and quantify some bioactive phytochemical constituents of *Nigella sativa* oil detected by HPLC.

**Name**	**Expected retention time**	**Retention time (min)**	**Area**	Amount (mg/kg)^*^
P hydroxybenzoic	6.55	6.70	129.9396	33.096
Catechin	7.00	6.91	7.1542	3.476
Chlorogenic acid	7.60	7.54	2.6632	0.832
Caffeic	8.40	8.33	16.0461	1.867
Syringic acid	9.40	9.54	20.4291	1.946
P Coumatic	10.65	10.71	248.7377	18.420
Rutin	12.20	12.14	3.7987	0.547
Ferulic	12.60	12.46	2.1443	0.812
O Cumaric	13.85	13.91	19.8253	1.167
Hesperidin	14.70	14.50	2.7104	0.547
Resveratrol	16.60	16.75	6.5367	1.897
Rosmarinic acid	16.90	16.97	3.4533	1.467
Myricetin	17.50	17.48	72.6164	8.667
Quercetin	18.80	18.88	14.8826	15.858
Apigenin	20.00	19.71	1.2579	0.019
Kaempferol	20.10	20.22	7.0725	0.988

^*^Three plant samples were used from the same area to obtain the mean.

**Table 3:** Identify and quantify some bioactive phytochemical constituents of *Nigella sativa* seed detected by HPLC.

**Name**	**Expected retention time (min)**	**Retention time (min)**	**Area**	Amount (mg/kg)^*^
Gallic	3.45	3.42	6.0037	0.597
Catechol	4.50	4.50	3.6324	1.196
P hydroxybenzoic	6.55	6.55	2.1215	0.540
Catechin	7.00	6.90	1.5636	0.760
Chlorogenic acid	7.6	7.47	31.09363	9.715
P Coumatic	10.65	10.60	34.6710	2.568
Rutin	12.20	11.96	12.2040	1.757
Ferulic	12,60	12.73	110.8616	41.987

^*^Three plant samples were used from the same area to obtain the mean.

### 3.3 Absolute and relative weight of the liver and kidney

All the experimental groups showed an insignificant impact on the absolute weight of the liver and kidney (Table 4). Meanwhile, the relative weight of both organs was significantly (*P* < 0.05) increased in the positive group administrated with AZA compared to the negative control group (3.23 ± 0.03 vs. 2.70 ± 0.15) g for the liver relative weight and (0.64 ± 0.03 vs. 0.50 ± 0.02) g for kidney relative weight, respectively. The treated groups received silymarin, NSO, and NSS with AZA normalized the liver and kidney relative weight to values nearest to the negative control group.

**Table 4:** Effect of NSO and NSS on absolute and relative weight of liver and kidney.

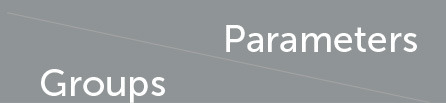	**Final body weight**	**Absolute liver weight (g)**	**Relative liver weight**	**Absolute kidney weight (g)**	**Relative kidney weight**
Saline negative control	276.2 ± 11.4	7.5 ± 0.9	2.70 ± 0.15	1.36 ± 0.16	0.50 ± 0.02
AZA positive control	242.3 ± 8.5	7.8 ± 0.5	3.23 ± 0.03^*^	1.56 ± 0.13	0.64 ± 0.03^*^
AZA+ Silymarin standard	270.8 ± 10.8	7.1 ± 1.1	2.59 ± 0.27	1.32 ± 0.18	0.49 ± 0.05
AZA + NSO	264.1 ± 12.4	6.8 ± 0.9	2.56 ± 0.28	1.30 ± 0.18	0.48 ± 0.04
AZA + NSS	258.8 ± 7.3	6.6 ± 0.8	2.57 ± 0.34	1.39 ± 0.15	0.52 ± 0.03
*P value*	<0.8654	<0.1483	<0.04459	<0.6495	<0.0480

The mark (^*^) in the same column is significantly (*P* < 0.05) comparable to the saline negative control. AZA, azathioprine; NSO, *Nigella sativa* oil; NSS, *Nigella sativa* seeds.

### 3.4 Lipid profile

**Figure 3:**
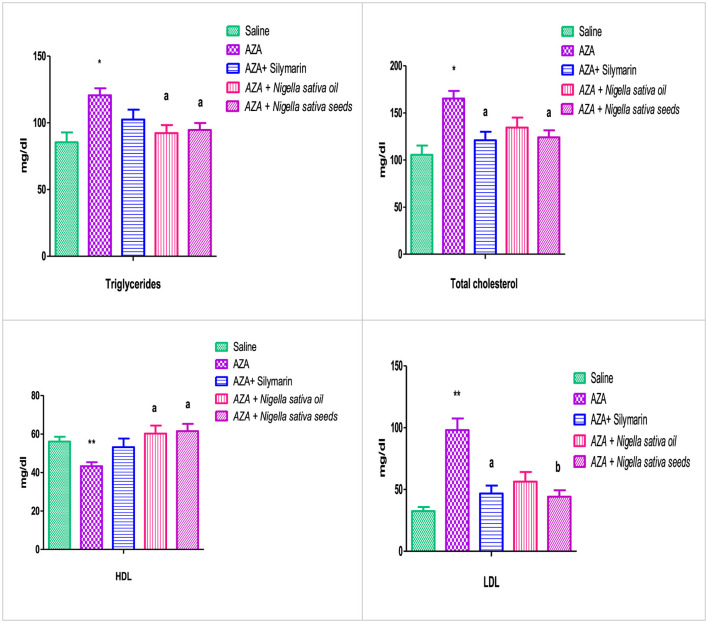
Effect of *Nigella sativa* oil and *Nigella sativa* seed on lipid profile (triglyceride, total cholesterol, HDL, and LDL). Bars denote means ± SE. The marks (*) and (**) are significantly *P* < 0.05 and *P* < 0.01 comparable to the saline negative control, respectively. The letters (a, b) are significantly *P* < 0.05 and *P* < 0.01 comparable to AZA (azathioprine) positive control, respectively.

The group that received AZA exhibited clear dyslipidemia compared to the negative control group, characterized by a significant increase in serum triglycerides and total cholesterol (*P* < 0.05), accompanied by a negative effect on serum HDL and LDL (Figure 3). There was a highly significant dropping in HDL and a rising increase in LDL (*P* < 0.01). NSO supplementation significantly (*P* < 0.05) mitigated serum triglyceride, and HDL but neither affected total cholesterol nor LDL. Whereas the group administered NSS showed significant (*P* < 0.05) improvement in serum triglyceride, total cholesterol, HDL, and LDL (*P* < 0.01) comparable to the AZA-positive group. The silymarin standard group exerted amelioration in serum total cholesterol, and LDL significantly (*P* < 0.05) with no effect on triglyceride, and LDL comparable to the AZA positive group.

### 3.5 Liver function

**Figure 4:**
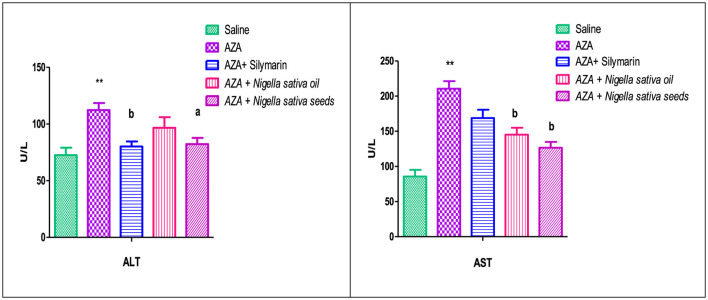
Effect of *Nigella sativa* oil and *Nigella sativa* seeds on liver function (serum ALT, alanine aminotransferase enzyme and AST, aspartate aminotransferase enzyme). Bars denote means ± SE. The mark (**) is significantly *P* < 0.05 comparable to the saline negative control. The letters (a, b) are significantly *P* < 0.05 and *P* < 0.01 comparable to AZA (azathioprine) positive control, respectively.

As shown in Figure 4, the AZA-treated group recorded a significant elevation in hepatic enzymes (ALT and AST) (*P* < 0.01) compared to the negative control group. There was a significant (*P* < 0.01) reduction in serum AST in groups that received both NSO and NSS. However, ALT recorded a significant (*P* < 0.05) decrease with NSS supplementation and an insignificant reduction in the NSO group compared to AZA positive group. The silymarin standard group showed a decrease in the liver enzymes, which was significant (*P* < 0.01) in ALT and insignificant in AST.

### 3.6 Kidney function

**Figure 5:**
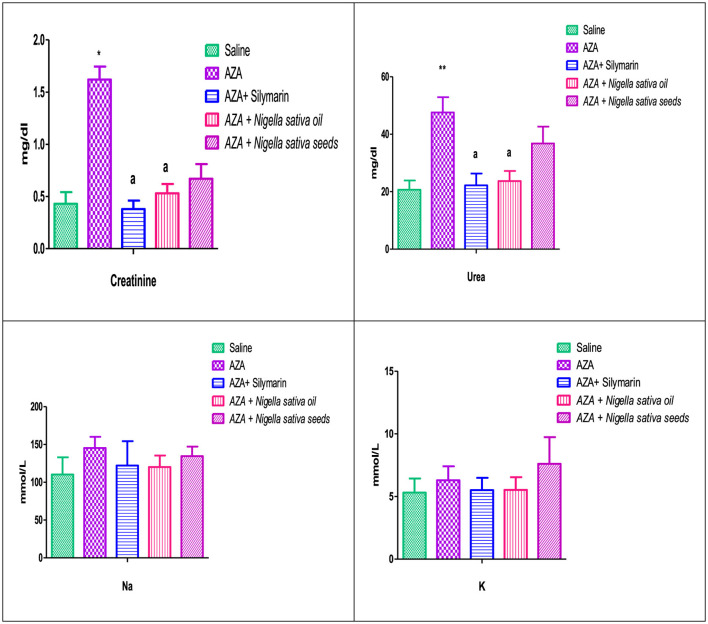
Effect of *Nigella sativa* oil and *Nigella sativa* seeds on kidney function (creatinine, urea, Na, and K). Bars denote means ± SE. The marks (*) and (**) are significantly *P* < 0.05 and *P* < 0.01 comparable to the saline negative control, respectively. The letter (a) is significantly *P* < 0.05 comparable to AZA (azathioprine) positive control.

Figure 5 illustrates a significant elevation in serum creatinine (*P* < 0.05), and urea (*P* < 0.01) as a response to AZA administration (positive control) related to the control negative group. Comparable to the AZA group, the silymarin standard group and rats received NSO significantly (*P* < 0.05) normalized serum creatinine and urea to a level nearest to the values of the control negative group. Unexpectedly, NSS showed an insignificant decrease in serum creatinine and urea. On the other side, Na and K did not exert any significant effect as responded by experimental treatments.

### 3.7 Inflammatory cytokines and cytokeratin18

**Figure 6:**
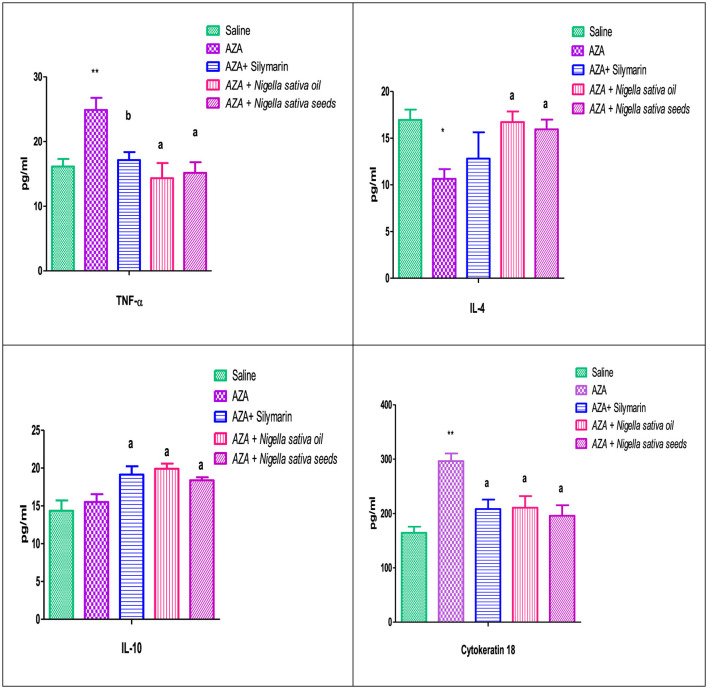
Effect of *Nigella sativa* oil and *Nigella sativa* seeds on cytokines (TNF-α, Tumar necrosis factor-α; IL-4, interlukine-4; IL-10, interlukine-10) and cytokeratin 18. Bars denote means ± SE. The marks (*) and (**) are significantly *P* < 0.05 and *P* < 0.01 comparable to the saline negative control, respectively. The letters (a, b) are significantly *P* < 0.05 and *P* < 0.01 comparable to AZA (azathioprine) positive control, respectively.

TNF-α, as a pro-inflammatory cytokine, showed a marked elevation in the positive control group that received AZA, significantly (*P* < 0.01) comparable to the negative control group. A significant reduction (*P* < 0.01) in TNF-α was achieved in the group given silymarin as a standard reference. On the other side, IL-4, and IL-10, as anti-inflammatory cytokines, were decreased in the serum of rats that received AZA comparable to the negative control; this decrease was significant (*P* < 0.05) for IL-4 and insignificant for IL-10 (Figure 6). The latter anti-inflammatory cytokine (IL-10) showed a significant (*P* < 0.05) increase in the silymarin standard group related to control positive administrated with AZA. Both NSO and NSS ameliorated the adverse effect of AZA in the inflammatory cytokines by downregulating TNF-α significantly and upregulating IL-4, and IL-10 (*P* < 0.05).

The serum cytokeratin 18 level were significantly increased (*P* < 0.01) with 15 mg/kg AZA treated group when compared to the control. In contrast, the silymarin standard group was able to modulate it significantly (*P* < 0.05). The administration of NSO and NSS reduced cytokeratin 18 levels significantly (*P* < 0.05) by nearly or even less than that obtained in the silymarin standard group (Figure 6).

### 3.8 Liver antioxidants and MDA

**Figure 7:**
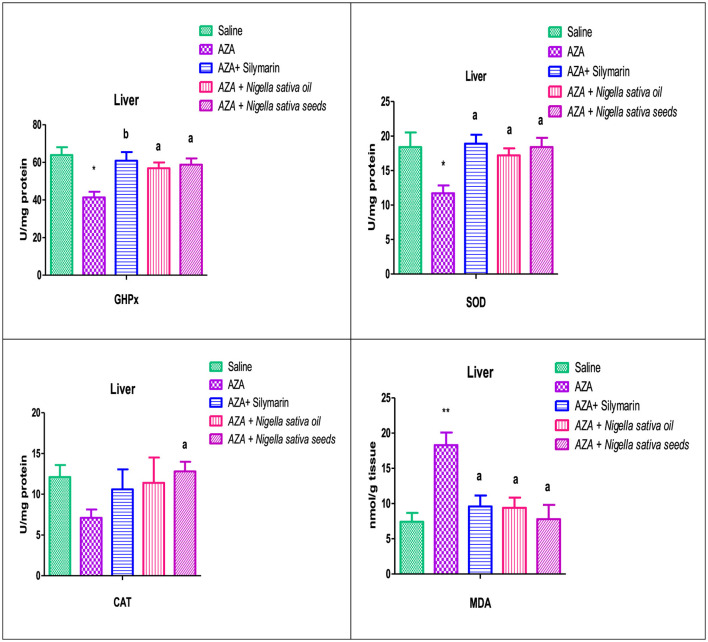
Effect of *Nigella sativa* oil and *Nigella sativa* seeds on liver tissue antioxidants (GHPx, glutathione peroxidase; SOD, superoxide dismutase; CAT, catalase) and lipid peroxidation (MDA, malondialdehyde). Bars denote means ± SE. The marks (*) and (**) are significantly *P* < 0.05 and *P* < 0.01 comparable to the saline negative control, respectively. The letters (a, b) are significantly *P* < 0.05 and *P* < 0.01 comparable to AZA (azathioprine) positive control, respectively.

The hepatic tissue of the AZA-treated group exhibited oxidative stress, indicated by a depletion of the activity of hepatic antioxidant enzymes, GHPx, SOD, and CAT. This was significant (*P* < 0.05) in GHPx and SOD, accompanied by a high significant (*P* < 0.01) elevation in hepatic MDA level (Figure 7). NSS supplementation significantly (*P* < 0.05) upregulated GHPx, SOD, and CAT with significantly downregulated (*P* < 0.05) in MDA as compared to the AZA group. The hepatic antioxidant enzymes GHPx and SOD for the NSS group were near the corresponding values of the silymarin standard group and superior in CAT. The same group (NSS) achieved the lowest value of the MDA compared with other experimental groups. Silymarin standard and NSO groups recorded an increase in the hepatic antioxidant enzymes which were significant (*P* < 0.05) in GHPx, (*P* < 0.05) in SOD, and insignificant in CAT with a significant drop in hepatic MDA (*P* < 0.05) in comparison with AZA-treated group.

### 3.9 Renal antioxidants and MDA

**Figure 8:**
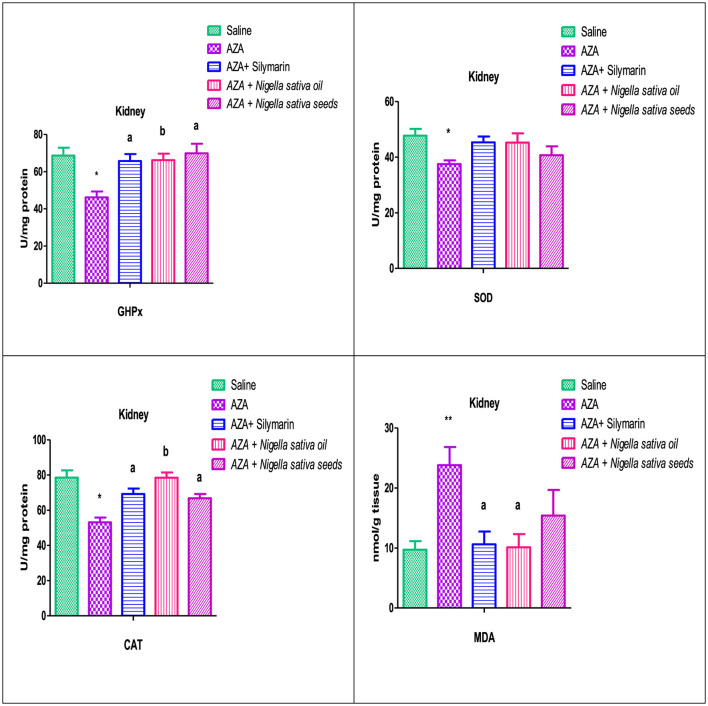
Effect of *Nigella sativa* oil and *Nigella sativa* seeds on kidney tissue antioxidants (GHPx, glutathione peroxidase; SOD, superoxide dismutase; CAT, catalase) and lipid peroxidation (MDA, malondialdehyde). Bars denote means ± SE. The marks (*) and (**) are significantly *P* < 0.05 and *P* < 0.01 comparable to the saline negative control, respectively. The letters (a, b) are significantly *P* < 0.05 and *P* < 0.01 comparable to AZA (azathioprine) positive control, respectively.

The group offered AZA supplementation exerted a significant decrease (*P* < 0.05) in the renal tissue antioxidant enzymes measured (GHPx, SOD, and CAT). In addition, there was a highly significant (*P* < 0.01) elevation in the renal tissue lipid peroxidation concentration represented by MDA compared to the control negative group (Figure 8). The Silymarin standard reference group, NSO, and NSS forms of NS exhibited a significant increase in renal antioxidant GHPx and CAT, which was at (*P* < 0.05) for silymarin and NSS and at (*P* < 0.01) for NSO comparable to the AZA-treated group. A considered increase was recorded in the renal antioxidant SOD in silymarin standard, NSO, and NSS groups. The MDA was significantly diminished (*P* < 0.05) by silymarin and NSO supplementations, while NSS had no significant effect on renal MDA.

### 3.10 Caspase-3 and caspase-9

**Figure 9:**
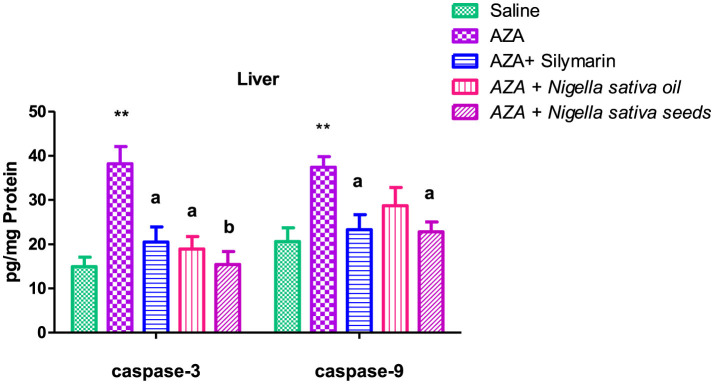
Effect of *Nigella sativa* oil and *Nigella sativa* seeds on liver tissue caspase-3 and caspase-9. The mark (**) is significantly different from the saline negative control at *P* < 0.05. Bars denote means ± SE. The mark (**) is significantly *P* < 0.05 comparable to the saline negative control. The letters (a, b) are significantly *P* < 0.05 and *P* < 0.01 comparable to AZA (azathioprine) positive control, respectively.

**Figure 10:**
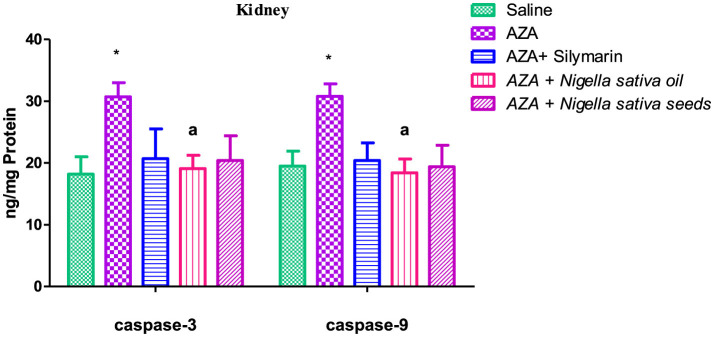
Effect of *Nigella sativa* oil and *Nigella sativa* seeds on kidney tissue caspase-3 and caspase-9. Bars denote means ± SE. The mark (*) is significantly *P* < 0.05 comparable to the saline negative control. The letter (a) is significantly *P* < 0.05 comparable to AZA (azathioprine) positive control.

Caspase-3 and caspase-9 levels were elevated in both liver and kidney tissues after AZA treatment, significantly (*P* < 0.01) for liver tissue and at (*P* < 0.05) for kidney tissue compared to negative control (Figures 9, 10). NSO and NSS supplementations for 6 weeks affected both caspase-3 and caspase-9 in liver and kidney tissues by various degrees as compared to the control positive group. NSS administration revealed significant rebalanced in caspase-3 (*P* < 0.01) and caspase-9 (*P* < 0.05) in the liver tissue (Figure 9). Meanwhile, NSO supplementation recorded significant improvement in liver caspase-3 (*P* < 0.05), at the same time the decrease in liver caspase-9 was insignificant. On the other side, NSO significantly (*P* < 0.05) normalized both caspase-3 and caspase-9 in the kidney tissue, while NSS supplementation had no significant effect on both caspase-3 and caspase-9 (Figure 10). The decrease of caspase-3 and caspase-9 in the silymarin standard group was significant (*P* < 0.05) only in liver tissue (Figure 9).

### 3.11 Histopathological examination

**Figure 11:**
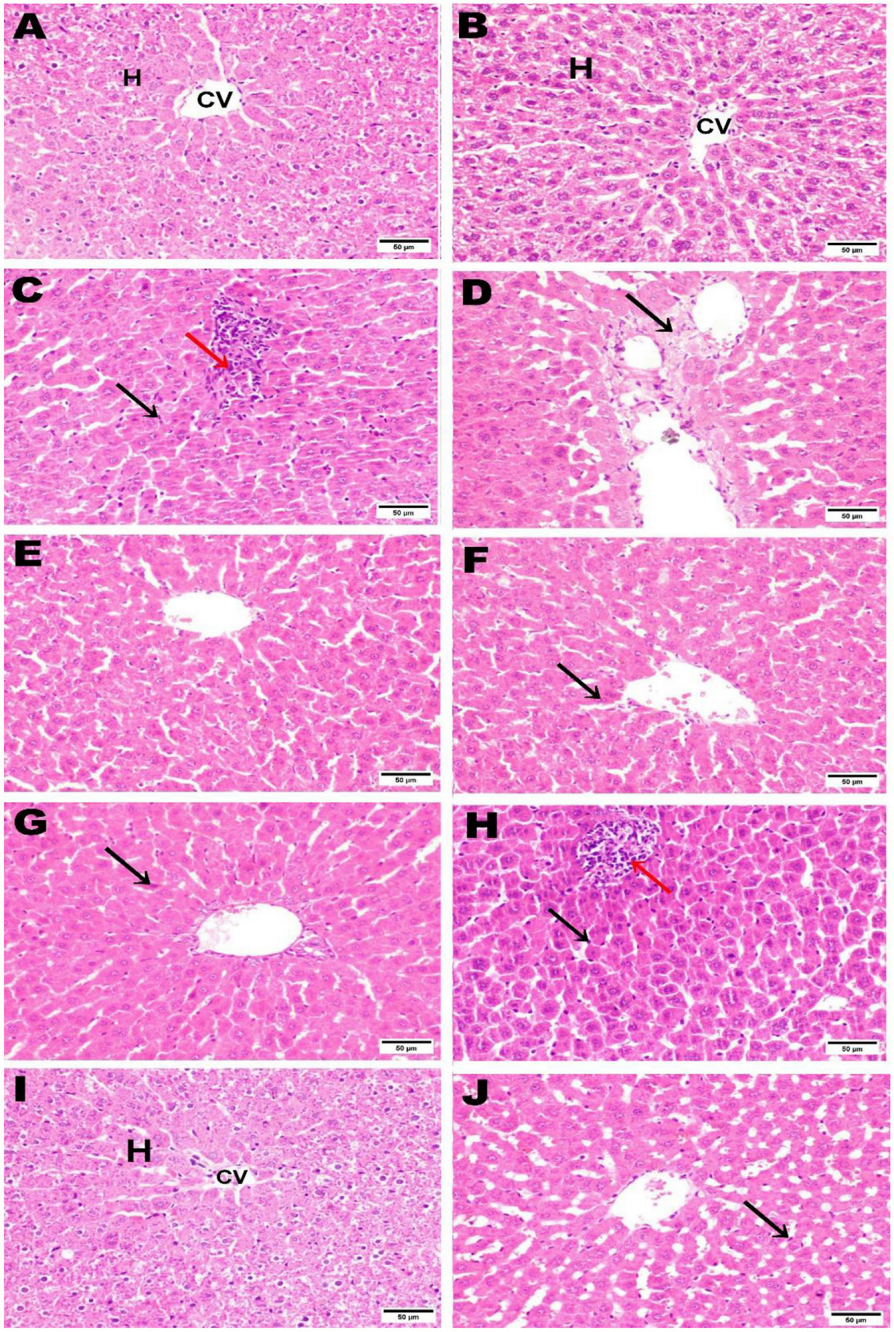
Hematoxylin and eosin (H&E; 400; scale bar, 50 m) were used to stain liver slices from each experimental group. The liver from the negative control **(A, B)** showed a normal photomicrograph of the hepatic lobule, including the central vein (CV) and hepatocytes **(H)**. The liver tissue from the AZA positive control revealed activation of Kupffer cells (black arrow) **(C)** and collagen fiber deposition around the central vein (black arrow) **(D)**. liver of rats administered silymarin showed no histopathological alterations **(E)** and few binucleations of hepatocytes (black arrow) **(F)**. liver of rats administered NSO demonstrated activation of Kupffer cells (black arrow) **(G)**, a small amount of localized hepatocellular necrosis, and a minor influx of inflammatory cells (red arrow) **(H)**. The NSS-treated group showed a typical hepatic lobule, with normal hepatocytes and central vein (CV) **(I)**. A few sections showed slight activation of the Kupffer cells (black arrow) **(J)**.

**Figure 12:**
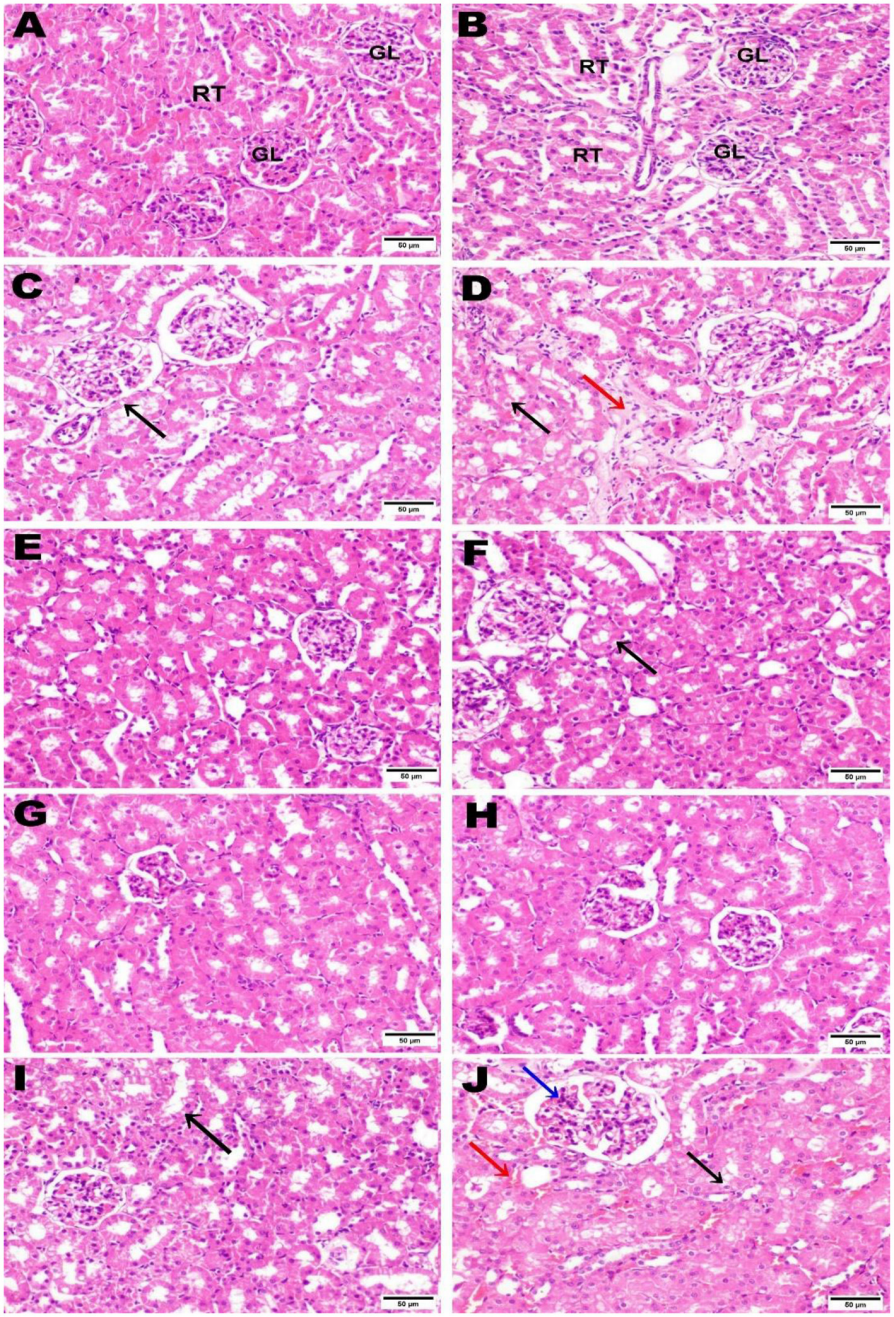
Hematoxylin and eosin (H&E; 400; scale bar, 50 m) were used to stain kidney slices from each experimental group. Kidney from negative control **(A, B)** illustrated normal histological architecture of renal parenchyma, including normal glomeruli (GL) and renal tubules (RT). The kidney from the AZA positive control revealed renal tubule epithelial vacuolar degeneration (black arrow) **(C)**, vacuolar degeneration of the epithelial lining of renal tubules (black arrow), and edema (red arrow) **(D)**. Kidney tissue from the group that received silymarin showed apparent normal renal parenchyma **(E)** and mild vacuolar degeneration of some renal tubule lining epithelial cells) **(F)**. Kidney of rats administered NSO demonstrated no histopathological alterations **(G, H)**. Most kidney sections of rats administered NSS showed slight vacuolar degeneration of some renal tubule lining epithelial cells (black arrow) **(I)**. In contrast, some sections showed congestion of intratubular capillaries (red arrow) with glomerular tuft (blue arrow) **(J)**.

The control negative group underwent microscopic examination, revealing the normal histological architecture of hepatic and renal tissues, evidenced by the normal hepatic lobule structure, normal central vein, and hepatocytes (Figures 11A, B), as well as normal renal parenchyma with intact glomeruli and renal tubules (Figures 12A, B). In contrast, the positive control group that received AZA showed significant liver and kidney injury, characterized by activation of Kupffer cells and localized hepatocellular necrosis with an influx of inflammatory cells and collagen fiber deposition around the central vein (Figures 11C, D), along with renal tubule epithelial vacuolar degeneration and interstitial edema (Figures 12C, D). The histological examination of liver and kidney tissues from rats that received silymarin, NSO, and NSS showed varying degrees of improvement compared to the positive control group. The silymarin standard group displayed no histopathological alterations in most liver sections, with only a few binucleations of hepatocytes observed in some areas (Figures 11E, F), and apparent normal renal parenchyma, although some renal tubules exhibited mild vacuolar degeneration (Figures 12E, F). Interestingly, the NSO group showed normal histological architecture in all renal sections examined (Figures 12G, H); meanwhile, this group also recorded activation of Kupffer cells and a small amount of localized hepatocellular necrosis associated with a minor influx of inflammatory cells (Figures 11G, H). Conversely, the liver tissue of rats supplemented with NSS exhibited a normal hepatic lobule structure, intact central vein, hepatocytes, and minimal activation of Kupffer cells in some sections (Figures 11I, J). In the NSS group, glomerular tuft congestion, intratubular capillary congestion, and mild vacuolar degeneration of certain renal tubule lining epithelial cells were observed (Figures 12I, J).

## 4 Discussion

Physiologically, the liver and kidneys are the most vital organs, performing numerous functions such as metabolism, detoxification, storage, and excretion of xenobiotics; thus, they are especially susceptible to damage. The liver and kidneys are closely related to each other. The liver is responsible for metabolizing harmful chemical substances, while the kidneys serve as the primary route for excreting metabolic waste. In addition, injured renal tissues release non-coding RNA into circulation, inducing remote hepatic injury. The high mortality associated with hepatorenal dysfunction underscores the need for natural interventions with fewer side effects to address this dysfunction. Herein, NSO and NSS were chosen for a comparative study to address a prevalent question: which form offers more potential protection and mitigates hepatorenal complications, considering the underlying mechanisms involving antioxidative, anti-inflammatory, and anti-apoptotic properties?

The significant increase in liver relative weight in the AZA-treated positive control group aligns with previous research (47). The elevation in liver relative weight observed in the AZA-supplemented group may be attributed to hepatocyte hypertrophy and extensive proliferation of collagen fibrils in the liver (47, 48). The present histological observations confirm this suggestion, which will be discussed later. The increase in relative kidney weight in the positive group that received AZA is due to interstitial edema and vacuolar degeneration of the epithelial lining of renal tubules, as observed in the present photomicrographic examination. Edema may explain the swelling and increased total organ weight observed during AZA intoxication (49). The normalization of liver and kidney relative weights in the treated groups with NSO and NSS suggests that both forms of Nigella sativa (oil and seeds) may mitigate hepatorenal injury induced by AZA.

The elevated dyslipidemia recorded in the AZA group, evidenced by a considerable rise in serum triglycerides and total cholesterol, a significant drop in HDL, and a significant increase in LDL, was a precursor to fatty liver and lipotoxicity, as previously suggested (50). These findings align with numerous studies indicating that AZA is a major cause of hyperlipidemia, particularly triglycerides (51). The hyperlipidemia observed in the current study is due to liver dysfunction because of oxidative stress caused by AZA (52). The explicit hypolipidemic effect observed with NSS matched recent research indicating that *Nigella sativa* seed administration markedly improves the lipid profile (53). The significant reduction in serum triglycerides and total cholesterol associated with NSO supports findings from Rashidmayvan et al., which reported a significant decrease in total cholesterol after NSO supplementation (54). Likewise, supplementation with NSO significantly reduced triglycerides and LDL while increasing HDL (55). Various theories have been proposed to explain the hypolipidemic effects of NS, suggesting that antioxidant constituents may inhibit cholesterol production in the liver and cholesterol absorption in the intestines (56). NS may improve energy metabolism in the mitochondria of liver cells, subsequently increasing fatty acid β-oxidation and preventing fatty liver (57).

A high elevation in hepatic enzymes (ALT and AST) observed with AZA administration aligns with previous work stating that serum ALT and AST levels in rats administered AZA were significantly elevated, indicating liver dysfunction (58). In patients on thiopurine therapy, elevated liver enzymes indicate the presence of liver damage (59). Notably, in the present study, the findings of hepatic enzymes obtained with AZA correlate with those of the lipid profile, confirming a significant positive association between liver enzymes and dyslipidemia (60). The increase in serum AST and ALT due to AZA causes inflammation, free radical damage, and cell damage that changes the permeability of hepatocyte membranes, leading to the release of AST and ALT into the bloodstream (61). NSS significantly mitigated the elevation of ALT and AST, a finding consistent with a previous study that stated NSS was effective in returning liver enzymes to normal levels via its anti-apoptotic and antioxidant abilities (53). In contrast, the effect of NSO on ALT and AST did not match that of NSS; it improved liver enzymes significantly for AST and insignificantly for ALT. However, one study found that NS supplementation only considerably lowered ALT levels (62). Another study found that those who consumed NS in the form of oil had significantly higher AST and marginally higher ALT levels (63). The potential benefits of NS are attributed to maintaining the structural integrity of the liver cell membrane and preventing hepatocyte destruction by reducing lipid peroxidation, thereby protecting against disruption caused by AZA (64). Nevertheless, there is a discrepancy regarding how NS affects liver enzymes, which may be due to the various forms of NS. Therefore, one of the targets of this study is to assess the two most popular forms of NS (oil and seed).

Estimation of creatinine, urea, Na, and K were assessed in the present study as key indicators of kidney function (65). The significant rise in serum creatinine and urea levels following AZA administration indicated renal dysfunction, as the impaired kidneys could not effectively eliminate creatinine and urea, leading to their accumulation in the bloodstream (66). These findings agree with previous work stating that AZA increased the concentrations of serum creatinine, urea, and uric acid (67). The rise in serum creatinine and urea in response to AZA is due to a reduction in glomerular filtration, as it impairs the antioxidant system and causes kidney impairment by inducing fibrosis, apoptosis, and inflammation (68). A significant suppression in serum creatinine and urea values achieved with NSO supplementation in this study indicates that NSO has a pronounced nephroprotective effect against AZA. NSO supplementation may restore alterations in kidney function due to the normalization of the glomerular filtration rate, which agrees with many researchers who studied the NSO form in their work (69). NSO supplementation exerted a mitigating effect on the levels of urea and creatinine (12, 18). In contrast to NSO, NSS failed to significantly lower creatinine and urea levels, suggesting a lower protective effect on the kidney. The non-significant decrease in serum electrolytes Na and K aligns with previous work (21). The nephroprotective effect of NSO recorded in the current study against AZA might have originated from the bioactive components of NS, such as alkaloids, saponins, vitamins, and minerals (12).

The present study relied on pro-inflammatory (TNF-α) and anti-inflammatory (IL-4 and IL-10) cytokines to investigate cytokine balance. The explicit elevation of TNF-α in the positive group receiving AZA was supported by many previous studies confirming the elevation of serum TNF-α and IL-6 in rats in response to AZA administration (70). AZA may stimulate inflammatory cells and cause an increase in the inflammatory response by releasing cytokines (71). These cytokines are responsible for the progression of damage to the liver (72) and kidney (73). The present results indicated that hepatorenal dysfunction was evident following AZA administration, as mentioned before (74). The organ damage due to azathioprine might originate from the destruction of mitochondrial membranes, causing inflammatory cytokine production and endothelial cell injury (73, 75). The present data demonstrates generalized tissue inflammation, as AZA reduced anti-inflammatory cytokines IL-4 and IL-10, which agrees with a previous study (76). The increase in pro-inflammatory cytokines over anti-inflammatory cytokines recorded in the present study confirms tissue inflammation, as previously mentioned (70, 77). NS in both forms used in the present study (oil and seeds) successfully mitigated the detrimental effects of AZA by downregulating TNF-α and upregulating IL-4 and IL-10. Interestingly, IL-4 and IL-10 levels were comparable to or even exceeded those in the silymarin-standard group. This finding aligns with recent studies that used *Nigella sativa* essential oil (78). It has been demonstrated that *Nigella sativa* L. can reduce organ damage and modify cytokines in various disorders, including liver and renal diseases (79). Pro-inflammatory cytokine levels (TNF-α and IL-6) were significantly decreased in rat serum and paw exudates by treatment with 400 mg/kg NSO (80). Likewise, the use of 10% NSO as a topical application in rats greatly ameliorated acute and subacute inflammation and reduced TNF-α levels by 50% (81). The present findings confirm that NS in both forms (oil and seeds) has anti-inflammatory action by suppressing the level of TNF-α and modulating IL-4 and IL-10, as reported in much research (79). Phenolic compounds, such as gallic acid esters, mainly derived from tannic acid, act as anti-inflammatory agents (82).

The present study used cytokeratin 18 as an accurate assessment of cell death and disease progression, as previously reported (83). Cytokeratin 18 is an intermediate filament protein that constitutes a major component in simple-type epithelial cells, including those in the liver, kidney, and other epithelial cells (84). The significant elevation in cytokeratin 18 concentration found in the AZA group indicated damage to the epithelial cells of the liver (59) and kidney (67). The damaged epithelial cells of the liver and kidney exhibited total cytokeratin-18 fragmentation during apoptosis by caspases, releasing it into circulation and highlighting the apoptotic cell death pathway, which causes damage to epithelial cells (85). The elevation of serum cytokeratin 18 concentrations was restored by administering both forms of NS. Indeed, cytokeratin 18 levels were reduced by the NSO form nearly to the levels observed in the silymarin standard group. Unfortunately, there is no existing work discussing the effect of NS supplementation on cytokeratin 18 concentrations. However, other therapeutic plants like NS have been cited for their pharmacological properties. Pomegranate fruit has significant antioxidant capacity and anti-inflammatory properties due to its polyphenols, which reduce cytokeratin 18 expression (86). *Cornus mas* L. fruit causes a significant decrease in cytokeratin 18 levels when treated for non-alcoholic fatty liver disease (87).

Reactive oxygen species originate from an imbalance between oxidant and antioxidant capacities pointing out oxidative stress in various tissues, especially in the liver, kidney, and brain (88). In the existing study, AZA treatment showed a significant depletion in the activity of hepatic GPx, SOD, and an insignificant reduction in CAT with a highly significant elevation in hepatic MDA level, which exerted severe oxidative stress induced by AZA that proved liver injury. These results were consistent with recent reports from various researchers (89). The significant reduction in renal tissue antioxidant enzymes GHPx, SOD, and CAT, along with an increase in renal MDA in response to AZA, evidenced renal damage, supporting previous work (90). The imbalance between the oxidant/antioxidant defense system is evident in the increased levels of MDA resulting from lipid peroxidation and the decrease in antioxidant enzymes observed in AZA-treated rats, confirming hepatorenal dysfunction (29). The hepatorenal damage observed with AZA is attributed to the conversion of AZA to 6-mercaptopurine and the accumulation of 6-methyl mercaptopurine metabolites in the cells due to glutathione depletion and mitochondrial injury, which causes organ toxicity (91). Meanwhile, the significant elevation in hepatic MDA levels was due to AZA-induced oxidative stress, triggering the generation of free radicals that harm liver and kidney cells by causing lipid peroxidation (29). The two forms of NS (oil and seeds) mitigated the perturbation of antioxidant enzymes caused by AZA intoxication in the liver and kidney to varying degrees: NSS supplementation had a predominant effect on liver antioxidants, significantly upregulating hepatic tissue GHPx, SOD, and CAT while significantly downregulating MDA. MDA levels were normalized when rabbits were offered *Nigella sativa* seeds (92). In contrast, NSO had a lesser effect, significantly increasing GHPx and SOD only in liver tissue while significantly decreasing MDA. Regarding renal antioxidants and lipid peroxidation, contrary to the results recorded in hepatic tissue, NSO showed a predominant effect, significantly rebalancing GHPx and CAT and demonstrating a significant reduction in MDA. NSS supplementation showed a non-significant decrease in MDA with improvements in GHPx and CAT. These findings were confirmed by previous studies indicating that *Nigella sativa*, particularly NSO, had a protective effect against chemotherapeutic drug-induced nephrotoxicity by suppressing lipid peroxidation and enhancing the activity of antioxidant enzymes in renal tissues (93). NSO treatment lowered signs of kidney damage by reducing tissue lipid peroxidation (12). Thymoquinone, the primary component of *Nigella sativa*, increased the effectiveness of human serum albumin in scavenging free radical ions (94).

In the existing study, caspase-3 and caspase-9 were chosen to determine apoptosis. The significant elevation of caspase-3 and caspase-9 in the AZA group was consistent with other studies reporting an increase in caspase-3 in the kidney after AZA treatment (58, 76). AZA treatment for 4 weeks elevated caspase-9 and subsequently induced apoptosis and tissue damage (70). The increase in apoptotic activity in liver and kidney tissues induced by AZA suggested tissue damage (95). A general benefit achieved by NSS and NSO was evidenced by the downregulation of apoptotic enzymes (caspase-3 and caspase-9) in liver and kidney tissues to varying degrees: NSS significantly improved caspase-3 and caspase-9 in liver tissue, consistent with a previous study indicating the antioxidant and anti-apoptotic properties of NSS help protect against hepatotoxicity induced by monosodium glutamate (51). Meanwhile, NSO recorded a partial improvement in caspase-9 in liver tissue, aligning with previous work that noted a statistically non-significant decrease in hepatic apoptosis with NSO supplementation (96). Conversely, the explicit anti-apoptotic effect of NSO in renal tissue observed in the present study was confirmed with normalized caspase-3 and caspase-9, supported by previous research reporting that nephrotoxicity caused by sodium nitrite was decreased by NSO via reducing inflammation, fibrosis, and oxidative stress, and inhibiting apoptosis by suppressing caspase-3 and pJNK/JNK (97). In addition, supplementation of 5 mL/kg body weight NSO exhibited a nephroprotective effect (98).

The histological examination of liver and kidney sections supported the notable data recorded in this study; the negative control illustrated normal histological observations, while the liver and kidney tissues of the positive control group that received AZA showed remarkable pathological damage to both organs, as previously reported (36, 58, 67). The alterations found in liver and kidney tissue in response to AZA might be due to oxidative stress, which alters cell membrane permeability, damages DNA, and leads to cell necrosis (96). Silymarin, NSO, and NSS showed varying degrees of improvement in examined liver and kidney tissues. The NSO group displayed normal histological architecture in all renal sections examined, indicating that NSO has a superior effect on renal tissue, supported by previous work showing significant preservation of both renal corpuscles and glomeruli and normal renal tubules after NSO administration (11). Meanwhile, the liver tissue of rats supplemented with NSS exhibited standard hepatic lobule histology, a healthy central vein, and hepatocytes, supporting that it is preferable to use NSS in liver complications. The normal lobular structure, hepatocytes, and portal tracts observed align with a study that found NSS restores liver tissue alterations (5).

Overall, the present data confirmed that AZA administration has an oxidative stress effect, demonstrating hepatorenal injury. *Nigella sativa* in both forms (NSO and NSS), with its total phenols and total flavonoids, possesses potential anti-inflammatory, anti-apoptotic, and antioxidant properties that underpin their protective capabilities against hepatorenal damage in response to AZA administration. NSO and NSS successfully mitigated the detrimental effect of AZA through their anti-inflammatory properties. The two forms downregulated TNF-α and upregulated IL-4 and IL-10. Interestingly, IL-4 and IL-10 levels were near or even superior to those of the silymarin standard group. The two forms of NS (oil and seeds) restored the perturbation of antioxidant enzymes caused by AZA intoxication in the liver and kidney to varying degrees: NSS supplementation had a predominant antioxidant effect in liver tissue, significantly upregulating hepatic tissue GHPx, SOD, and CAT while downregulating MDA. NSO had a predominant antioxidant effect in renal tissue, significantly rebalancing GHPx and CAT and suppressing lipid peroxidation. The anti-apoptotic properties of NSS and NSO were evidenced by the downregulation of caspase-3 and caspase-9 in liver and kidney tissues to varying degrees: NSS significantly inhibited apoptosis by reducing caspase-3 and caspase-9 in liver tissue. Conversely, the explicit anti-apoptotic effect of NSO in renal tissue was established with normalization of both caspase-3 and caspase-9.

These pharmacological properties of *Nigella sativa* are attributed to the bioactive phytochemical constituents detected by HPLC. Notably, NSO and NSS ameliorated hepatorenal injury, evidenced by the previously mentioned results of liver and kidney function tests, lipid profiles, inflammatory cytokines, cytokeratin 18, antioxidants in both organ tissues, anti-apoptotic effects, and histological observations. An in-depth analysis of the previous data shows that NSO normalized oxidative damage in the kidney in response to AZA, which surpassed that of NSS. Conversely, the protection of liver injury with NSS supplementation was superior to that of NSO. These interesting observations may be attributed to the significant differences in bioactive phytochemical constituents of NSO and NSS detected in this study. Ferulic acid, a major component of NSS, may protect the liver from injury, as previously reported (97). Recent studies suggest that ferulic acid is an effective agent for preventing oxidative damage to liver tissue in non-alcoholic fatty liver disease (98). Ferulic acid reduces oxidative stress, hepatic inflammation, and fibrotic responses in the liver through PTP1BAMPK signaling pathways (99). P-hydroxybenzoic acid, p-Coumaric acid, and quercetin were the abundant bioactive constituents detected by HPLC in NSO, contributing to reduced inflammatory processes and oxidative stress in renal tissue. P-hydroxybenzoic acid has therapeutic advantages as an antioxidant (100) and acts against inflammation (101). Quercetin demonstrates bioactive characteristics linked to the amelioration of cell damage and alleviates symptoms of renal failure (102). Quercetin protects against changes in glomerulus ultrastructure, reduces blood levels of urea and creatinine, and lowers oxidative stress and biomarkers of inflammation in a rat model of renal failure (103).

## 5 Conclusion

The existing study proved that AZA treatment induced hepatorenal injury; nevertheless, supplementation of NSO and NSS mitigated this adverse effect to varying degrees through their antioxidant, anti-inflammatory, and anti-apoptotic properties, which underlie their protective performance against hepatorenal damage in response to AZA administration. An in-depth analysis of the study's overall findings, particularly those related to liver enzymes, creatinine, urea, lipid profile, antioxidant enzyme activities, and caspase-3 and caspase-9 levels in liver and renal tissues, concludes that the administration of NSO surpassed that of NSS in restoring oxidative damage to the kidney. On the other hand, NSS provided better protection against liver injury than NSO. This interesting observation suggests recommending NSO for patients suffering from kidney dysfunction while using NSS for those with liver problems.

## Data Availability

The raw data supporting the conclusions of this article will be made available by the authors, without undue reservation.
